# Conformational ensembles of an RNA hairpin using molecular dynamics and sparse NMR data

**DOI:** 10.1093/nar/gkz1184

**Published:** 2019-12-31

**Authors:** Sabine Reißer, Silvia Zucchelli, Stefano Gustincich, Giovanni Bussi

**Affiliations:** 1 Scuola Internazionale Superiore di Studi Avanzati (SISSA), Via Bonomea 265, 34136 Trieste, Italy; 2 Department of Health Sciences, Center for Autoimmune and Allergic Diseases (CAAD) and Interdisciplinary Research Center of Autoimmune Diseases (IRCAD), University of Piemonte Orientale, Novara, Italy; 3 Central RNA Laboratory and Department of Neuroscience and Brain Technologies, Istituto Italiano di Tecnologia (IIT), 16163 Genova, Italy

## Abstract

Solution nuclear magnetic resonance (NMR) experiments allow RNA dynamics to be determined in an aqueous environment. However, when a limited number of peaks are assigned, it is difficult to obtain structural information. We here show a protocol based on the combination of experimental data (Nuclear Overhauser Effect, NOE) and molecular dynamics simulations with enhanced sampling methods. This protocol allows to (a) obtain a maximum entropy ensemble compatible with NMR restraints and (b) obtain a minimal set of metastable conformations compatible with the experimental data (maximum parsimony). The method is applied to a hairpin of 29 nt from an inverted SINEB2, which is part of the SINEUP family and has been shown to enhance protein translation. A clustering procedure is introduced where the annotation of base-base interactions and glycosidic bond angles is used as a metric. By reweighting the contributions of the clusters, minimal sets of four conformations could be found which are compatible with the experimental data. A motif search on the structural database showed that some identified low-population states are present in experimental structures of other RNA transcripts. The introduced method can be applied to characterize RNA dynamics in systems where a limited amount of NMR information is available.

## INTRODUCTION

RNA plays a fundamental role in the cell. It encodes the amino acid sequence of proteins (messenger RNA, mRNA) (1), is used as an adapter in translation (transfer RNA, tRNA) (2) and performs protein synthesis (ribosomal RNA, rRNA) (3). In addition, in the last decades a growing number of non-coding RNAs have been discovered playing important roles in regulation (4,5). RNA function is often linked to its conformational dynamics rather than to a unique structure (6,7). Extreme examples in this sense are riboswitches (8), that can adopt different, competing metastable structures whose relative stability is controlled by the cellular environment. Advanced nuclear magnetic resonance (NMR) techniques, and in particular relaxation dispersion methods (9), provide a powerful approach to the identification of so-called ‘excited states’ in solution and have been used to identify transient states in RNA (10).

Recent studies reported the capability of a non-coding antisense RNA from an inverted SINEB2 to control and, in particular, increase protein synthesis (11–15). A later structural analysis of a functionally important, terminal hairpin from this RNA has reported solution NMR data that allowed for the three-dimensional structure of this terminal hairpin to be solved (16). However, it was not possible to find one structure which was in agreement with the entire set of all Nuclear Overhauser Effect (NOE) data at once. It is therefore likely that the NMR data represent an average over distinct conformations with mutually exclusive NOE signals.

Molecular dynamics (MD) simulations in principle provide a powerful tool to access RNA dynamics at virtually unlimited space and time resolution, and can be performed in an environment very similar to that used in NMR. As such, they are perfectly complementary to lower-resolution NMR approaches (17). However, their result is often not satisfactory mostly due to the short accessible time-scales and the inaccuracy of the employed force fields. The former issue can be tackled using enhanced sampling methods (18,19). The latter is usually addressed by complementing MD with experimental data (20). A formalism that emerged in the last years is the so-called maximum entropy (ME) approach (21), where the structural ensemble generated by an imperfect force field is minimally modified in order to fit a set of experimental data (see (22) for a recent review). The ME procedure allows to generate ensembles that are by construction in agreement with experimental data. A different strategy is used in so-called maximum parsimony (MP) approaches, where, in agreement with Occam’s razor principle, a minimal number of conformations required to explain experimental data is retained (23,24).

In this paper, we use an MD approach applying state-of-the-art enhanced sampling techniques and an ME method to obtain an ensemble compatible with all NOE data. To get a better understanding of the type of conformations required to satisfy the NOE data, the ensemble structures were clustered and NOE data were back-calculated for each cluster. Then, the contributions of the clusters were reweighted to find a minimal set of clusters sufficient to explain the experimental data, following the MP approach. We find that 4 clusters with different base-pairing pattern at least are necessary to explain the experimental data. Finally, we performed a motif search on the PDB finding that structural patterns similar to those of the essential metastable conformations were already observed in other RNA transcripts.

## MATERIALS AND METHODS

### Simulation settings

Simulations were performed with GROMACS 5.1.2 (25), using TIP3P water (26), the AMBER force field for nucleic acids (AMBER99 + PARMBSC0 + χ_*OL*3_) (27–29), and ion parameters from Joung and Cheatham (30). Although some improvements have been recently proposed (31–33), this force field is to date the most validated one for RNA simulations (32). The stochastic velocity rescaling thermostat (34) was used to keep the system at a temperature of 298 K in combination with the Berendsen barostat to keep the pressure at 1 bar (35). The system had 57 494 atoms, 928 of which constitute the solute; the rest were 64 sodium ions, 36 chloride ions and 6274 water molecules, resulting in a neutralized system with a salt concentration of 0.1 mol/l.

#### Unrestrained simulation

The first model of the NMR-refined structure (PDB #5lsn (16)) was used as a starting structure for an unrestrained 600 ns MD simulation. Atom coordinates were saved every 10 ps.

#### Replica exchange with collective-variable tempering (RECT)

MD simulations were performed using a RECT scheme (36) with eight replicas. In this replica-exchange approach, one replica is unbiased and analyzed whereas the other replicas are progressively biased in order to increase sampling of *a priori* chosen degrees of freedom. Enhanced sampling simulations were performed using the PLUMED plugin (37). The eight starting structures have been selected randomly from the last 400 ns of the equilibration trajectory and share the same basic arrangement, with some showing additional off-register non-canonical base pairs in the stem with respect to the deposited pdb models, see Supplementary Figure S1. To increase sampling of the loop region, biasing potentials were added on the glycosidic bond angles and on the coordination number between the centers of mass of nucleobases 14, 15 and 16 and the centers of mass of all the nucleobases. Bias factors followed a geometric progression along the replica index up to a maximum value equal to 8. More details about the enhanced sampling parameters are reported in Supplementary Material.

### NOE restraints

The NOE data were taken from (16) and consisted of a list of 125 maximum and minimum interprotonic distances. The maximum distances in the list are 3.6 Å for strong signals, 5.0 Å for medium signals and 6.5 Å for weak signals. For this study, the minimum distances were ignored. Given the assumption that the conformational dynamics are much slower than the molecular tumbling, the NOE forward model can be approximated by

$$\begin{equation*} f(d_i) = f_{{\rm NOE}}(d_i) = 1/d_{i}^{\, 6}, \end{equation*}$$

with *d* being the absolute distance between the atoms *j* and *k* and *i* ∈ [1, 125] being the index of the given proton pair *jk* (38,39).

The NMR distances were used as restraints on the ensemble average, to obtain an ensemble in which

$$\begin{equation*} \langle f (d_i) \rangle _{e} = \frac{1}{T}\ \sum _{t=1}^T f (d_{i,t}) \ge f(d_{i,{\rm exp}}) \end{equation*}$$

for all restraints, where *T* is the total number of snapshots in the ensemble.

Restraints on the NOE ensemble averages 〈*f*(*d*_*i*_)〉_*e*_ were applied according to the maximum entropy principle using the PLUMED plugin and described in detail in (40). Using Lagrangian multipliers λ_*i*_, an additional potential is applied to correct the ensemble average. Since we wanted to ensure lower limits of 〈*f*(*d*_*i*_)〉_*e*_, inequality restraints were used, which means that the correcting potential is only applied if λ_*i*_ < 0 (as described in Supplementary information of (40)). A Gaussian prior for the error was also included with σ = 0.5 nm^−6^. λ_*i*_ were scaled along with the replica index, from 1 in replica 1 to 0.1 in replica 8, following a geometric progression. More details about the NOE restraints are reported in Supplementary Material. A sample input file is included in the Supplementary Material and in the PLUMED-NEST repository (41) as plumID: 19.072.

### Reweighting

In principle, converged restrained simulations would result in averages compatible with experiments. In practice, to obtain ensemble averages compatible with the experimental restraints without the need to fully converge the calculation, snapshots were reweighted using the ME approach as described in (22), such that the weighted average 〈*f*(*d*_*i*_)〉_*we*_ satisfies

$$\begin{equation*} \langle f(d_{i})\rangle _{we} = \sum _{t=1}^T\ w_t\ f(d_{i,t}) \ge f(d_{i,{\rm exp}}) \end{equation*}$$

for all restraints *i*, where $w$_*t*_ is the weight for snapshot *t*, with $\sum _{t=1}^T w_t = 1$.

The weighted median med[*f*(*d*_*i*_)]_*cl*_ is defined as the maximum signal in the cluster, for which the sum of weights of that value and all lower signals is <0.5 of the sum of weights of all snapshots in the cluster.

#### Statistical efficiency

The statistical efficiency was quantified using the normalized Kish’s effective sample size (42) defined as

$$\begin{equation*} s = \langle w_t \rangle _T^2/\langle w_t^2\rangle _T \end{equation*}$$

*s* can have values between zero and one and is equal to one if all weights are equal (all $w$_*t*_ = 1/*T*).

### Clustering

To find clusters of mutually similar conformations, the eRMSD (43) and the glycosidic bond angles χ were used to compare pairs of structures. For the pre-clustering, the χ angle of each residue was translated into a bit in order to force conformations with different *syn*/*anti* pattern to be assigned to different clusters. The bit was 1 if 0 < χ < 115° (*syn* conformation) and 0 otherwise (*anti* or *high-anti* conformation). The χ conformation of a snapshot is the sequence of the 29 bits obtained for all 29 nucleotides. All snapshots with the same χ conformation were put into the same pre-cluster.

The pre-clusters were then further clustered using the eRMSD as a similarity measure. PLUMED was used to calculate the eRMSD between all pairs of snapshots *t* and *t*′. A neighbor matrix $\mathbf {M}$ with dimensions *Q* × *Q* (*Q* is the number of snapshots per pre-cluster) was calculated with entries $M_{tt^{\prime }}=1$ if the eRMSD between the two structures *t* and *t*′ was <0.7, otherwise $M_{tt^{\prime }}=0$. Based on $\mathbf {M}$ and taking into account the weight for each snapshot obtained from the reweighting, the pre-clusters were clustered using a maximum clique search algorithm (44,45). Using this algorithm, all structures within a cluster are guaranteed to have pairwise eRMSD <0.7 and identical χ conformation. This choice allows the clusters to be conformationally homogenous and easy to interpret from a structural point of view.

The population of a cluster *c* is the sum of the weights of its members, *P*_*c*_ = ∑_*t* ∈ *c*_ $w$_*t*_.

### Minimal set of clusters

The weighted average signals for all clusters *c* with *P*_*c*_ > 0.002 were calculated as

$$\begin{equation*} \langle f(d_{i}) \rangle _{wc} = \frac{\sum _{t\in c}\ w_t\ f(d_{i,t})}{\sum _{t\in c} w_t} \end{equation*}$$

A grid search was performed to find a minimal set of *Y* clusters which could be reweighted such that all average signals are above the thresholds. *Y* was initialized to 1 and increased until at least one set was found. The search was performed by assigning all possible combinations of *Y* weights $w^{\prime }_y \in [0.01,(N-Y+1)\cdot 0.01]$, with $\sum\nolimits _{y=1}^Y w^{\prime }_y = 1$ (e.g. for *Y* = 4, one combination is $w^{\prime }_{1}=0.01, w^{\prime }_{2}=0.45,w^{\prime }_{3}=0.32,w^{\prime }_{4}=0.22$), with *N* = 100.

The averages signals for each combination of weights are

$$\begin{equation*} \langle f(d_{i}) \rangle _{\text{set}} = \sum _{y\in \text{set}}^Y\ w^{\prime }_y \langle f(d_{i}) \rangle _{wy} \end{equation*}$$

For each set for which all average signals were above the thresholds, the Kullback–Leibler divergence between the original populations of the clusters and the new weights $w^{\prime }_{y}$ was calculated to identify which combination of clusters was leading to an ensemble more similar to the original one.

$$\begin{equation*} D_{KL}(w^{\prime }_y||P_y)=\sum _{y\in \text{set}}\, w^{\prime }_{y}\, \text{ln}\frac{w^{\prime }_{y}}{P_{y}} \end{equation*}$$

### Annotations

The interaction annotations (upward, downward, inward and outward stackings, base pairs according to the Leontis–Westhof classification (46)) between all pairs of bases were calculated for each snapshot using *Barnaba* (47).

### Secondary structures

The annotations for each cluster were visualized using the dynamic secondary structure representation from *Barnaba* (47). The colors scale goes from yellow (annotation present in 10% of all structures in the cluster) to black (annotation present in 100% of all structures in the cluster).

### PDB motif search

All RNA-containing structures from the PDB database dated 4 October 2019, have been subject to a motif search to find structurally similar, sequence independent motifs, where the query and the target are as similar as eRMSD <1.0. For this, the hairpin has been reduced to the loop region, i.e. the nine central residues #11 to #19. The number of residues included in the search has been reduced when compared with the 13 residues used in (16) in order to allow for a matching of the loop structure also in presence of shorter stems. First the centroid structure with the minimum mean square eRMSD to all other structures in the simulated ensemble (RECT) has been identified. Then, the triangular equality in the definition of the eRMSD has been used: the motif matches for all structures in the simulated ensemble below the eRMSD threshold of 1.0 can be found by performing a motif search on the centroid, using as eRMSD threshold the maximum pairwise distance between the centroid and any structure from the ensemble, plus 1.0. The final matches for the individual structures from the simulated ensemble have been obtained by running an eRMSD calculation between all matches and the simulated trajectory, and then taking all matches with eRMSD <1.0.

The χ conformations for all matches have been calculated, and only matches with the same χ conformation as the query were kept. The distances between atom O3’ of residue *i* and P of residue *i* + 1 have been checked to verify that all residues are connected on a single strand.

The matches in ribosomal structures have been annotated with *Barnaba* to find basepairs between the loop and other parts of the ribosome. The 9 nt loop match had to have at least 3 bp toward the exterior, which was defined as any residue outside the loop plus/minus 10 residues. The exterior basepair-forming nucleotides had to be on one strand, within maximum 10 residues distance to each other, and basepairs had to be conserved when replacing the loop sequence with the corresponding SINEB2 loop sequence.

## RESULTS

### NMR-refined structure

The NMR-refined structure of the terminal hairpin SL1 of the inverted SINEB2 transcript (PDB #5lsn (16)) contains ten models which have essentially identical conformations, with exception of residue 16 (in the PDB numbering residue 79), which has a glycosidic bond angle χ_16_ of either ≈100° (seven models, set NMR1) or ≈40° (three models, set NMR2), both corresponding to a rare *syn* conformation. This results in a stacking of residue 15 on residue 14 in set NMR1 (Supplementary Figure S1). The deposited 125 proton distance thresholds can be classified in three groups: short (s) with a maximum distance 3.6 Å, medium (m) with a maximum distance 5.0 Å and long (l) with a maximum distance 6.5 Å. Set NMR1 has eight violations of the distance thresholds, two in the terminal region (3U_H6_–2C_H1′_ and 26G_H8_–25U_H6_), one in the middle of the double strand (8G_H8_–9U_H6_), and five close to or within the loop region (11G_H8_–12U_H6_, 14G_H8_–13U_H1′_, 14G_H8_–14G_H1′_, 15U_H6_–15U_H1′_, 17A_H8_–16G_H8_). Set NMR2 has an additional violation related to the rotated base 16 (17A_H8_–16G_H1′_). The relative average signals 〈*f*(*d*_*i*_)〉_*e*_/*f*(*d*_*i*,exp_) of the violations are listed in Table 1, columns NMR1 and NMR2, in bold numbers (i.e. 〈*f*(*d*_*i*_)〉_*e*_/*f*(*d*_*i*,exp_) < 1). From the number of violations it can be assumed that either, the models in the deposited structure do not represent the optimal ground-state, or, that there are additional, metastable conformations which have significant contributions to the NOE signals.

**Table 1.** Critical NOEs with 〈*f*(*d*_*i*_)〉_*e*_/*f*(*d*_*i*,exp_) < 1.2 in at least one of the ensembles. If the value is <1, the threshold is violated (in **bold**). NMR1 are the 7 models with χ_16_ = 100° deposited in the PDB database (#5lsn), NMR2 are the remaining 3 models with χ_16_ = 40°. MD is the ensemble from the unrestrained simulation. RECT is the ensemble from the enhanced sampling simulation with ME restraints. *d*_exp_ are the experimental maximum distances, s (short) = 3.6 Å, m (medium) = 5.0 Å, l (long) = 6.5 Å

Pair	*d*_exp_	$\frac{\langle f(d_{i}) \rangle }{f_{i,{\rm exp}}}$			
		NMR1	NMR2	MD^a^	RECT^b^
2C_H6_–1C_H1′’_	m	1.24	1.24	1.18	1.38
3U_H6_–2C_H1′_	m	**0.97**	**0.97**	1.28	1.10
4C_H6_–3U_H1′_	m	1.18	1.18	1.06	2.23
6U_H6_–5G_H1′_	m	1.02	1.02	1.06	1.04
8G_H8_–9U_H6_	m	**0.98**	**0.99**	1.12	1.11
11G_H8_–10G_H1′_	m	1.17	1.16	1.33	1.29
11G_H8_–12U_H6_	m	**0.96**	**0.96**	**0.95**	1.14
12U_H6_–11G_H1′_	m	1.20	1.21	1.02	**0.94**
14G_H8_–13U_H1′_	m	**0.96**	**0.95**	**0.68**	2.39
14G_H8_–14G_H1′_	s	**0.94**	**0.95**	**0.73**	2.45
14G_H8_–15U_H6_	l	2.12	1.10	2.12	1.62
15U_H6_–15U_H1′_	s	**0.88**	**0.87**	**0.88**	1.16
17A_H2_–16G_H1′_	m	1.75	1.89	1.64	1.19
17A_H8_–16G_H1′_	l	5.01	**0.99**	4.03	8.70
17A_H8_–16G_H8_	m	**0.88**	**0.99**	**0.46**	**0.54**
17A_H8_–18A_H8_	m	1.06	1.60	3.04	2.06
18A_H8_–19C_H6_	m	1.04	1.22	**0.88**	1.16
19C_H6_–18A_H1′_	m	1.15	1.14	1.11	4.17
20C_H6_–19C_H1′_	m	1.19	1.18	1.23	1.23
22C_H6_–21A_H1′_	m	1.20	1.20	1.15	1.15
25U_H6_–24A_H1′_	m	1.07	1.05	**0.97**	**0.95**
26G_H8_–25U_H6_	m	**0.98**	**0.98**	1.16	**0.99**
29G_H8_–28G_H1′_	m	1.34	1.33	1.16	1.40

^a^Snapshots with *dt* = 4 ps.

^b^Snapshots with *dt* = 100 ps.

### Unrestrained MD simulation

An MD simulation was performed to check if agreement with experimental thresholds could be improved by averaging the signal over a trajectory sampled in a simulation box with explicit water and ions, instead of a set of 10 individually refined conformations. No rearrangements of the loop region took place during the course of the 600 ns simulation, as it can be seen in the evolution of χ_14_, χ_15_ and χ_16_ in Supplementary Figure S2. Again, the relative average signals 〈*f*(*d*_*i*_)〉_*e*_/*f*(*d*_*i*,exp_) have been calculated to identify violations. The violations for the five hydrogen pairs close to or within the loop region present in the NMR structures are also present in the MD ensemble averages, see Table 1, column MD. Additionally, another NOE close to the loop (18A_H8_–19C_H6_) and one close to the GU pair at 5–25 (25U_H6_–24A_H1′_) were violated. The remaining violations seen in the NMR structure have disappeared, including the violation related to the χ_16_, which has an average value of 72°.

The overall number of violations is lower in the MD ensemble with respect to the NMR ensembles, suggesting that the dynamics close to the native state and the accuracy of the force field better elucidate the experimental data. However, the remaining MD violations are typically further from the experimental value, suggesting that the individual NMR structures are overrestrained in order to comply simultaneously with all the experimental datapoints. Two of the violated NOE observables, pairs 14G_H8_–14G_H1′_ and 15U_H6_–15U_H1′_ are directly related to the glycosidic bond angles χ in the respective residues 14 and 15. If χ is in the *syn* conformation (χ ≈ 60°), the typical distance between H1′ on the sugar and H8/H6 (purines/pyrimidines) of the base of the same residue is ≈2.5 Å, see Supplementary Figure S3. In the *anti/high-anti* conformations (115° < χ < 360°), the typical distance is ≈3.7 Å. Since both pairs 14G_H8_–14G_H1′_ and 15U_H6_–15U_H1′_ have short distance thresholds with a maximum distance of 3.6 Å, it is likely there is some population of *syn* for residues 14 and 15.

### RECT combined with adaptive maximum entropy restraints

Since most violated NOE restraints are in the loop region, and because of the relationship between the χ angles and the intra-molecular NOEs in the loop region, the sampling in the loop was enhanced using a RECT scheme with 8 replicas, in which the bias potential on χ_14_, χ_15_ and χ_16_ was gradually increased. To enforce the agreement of the NOE observables with the experimental data, adaptive restraints according to the ME principle were applied using all 125 NOE distances as restraints. The simulation ran for 607 ns per replica and the unbiased replica was used for further analysis. We recall that when using inequality restraints to enforce a signal at least as large as the experimental one, the prior distribution given by the force field is only affected when Lagrangian multipliers are negative (Supplementary information of (40)). Only for four NOEs, the Lagrangian multipliers λ_*i*_ where significantly below zero, i.e. correcting potentials to shift the ensemble averages 〈*f*(*d*_*i*_)〉_*e*_ upward to their experimental thresholds were applied (Supplementary Figure S4). These NOE pairs were again in the loop region (14G_H8_–13U_H1′_, 14G_H8_–14G_H1′_, 15U_H6_–15U_H1′_ and 17A_H8_–16G_H8_) and are the ones which were strongest violated in the unrestrained MD, as expected. While the first three have λ_*i*_ < 0 in the first part of the simulation and then reach positive values (i.e. have correct ensemble averages and do not require a correcting potential any more), $\lambda _{17A_{H8}{-}16G_{H8}}$ continuously decreases during the entire runtime of the simulation. Additionally to the missing convergence of the Lagrangian multipliers, also the replica exchange scheme does not converge within the simulated time, which can be seen by the relative populations of the single replica in Supplementary Figure S5, which should be constant for all replica in a converged simulation.

The NOE violations of the ensemble averages decreased during the simulation, as shown by the black curve in Supplementary Figure S6. The minimum ensemble violation (definition of violations in the Supplementary Material) is 0.35 reached at 220 ns, then it continuously increases to a final value of 0.55 (for comparison the ensemble violation of the unrestrained MD simulation was 1.51). The instantaneous violation of individual snapshots is generally one order of magnitude larger than the ensemble violation and ranges from 2 to 20, and increases and fluctuates more as the simulation continues. This is largely due to the increased heterogeneity of the ensemble.

In order to make the analysis less computational expensive, snaphots were selected with a stride of 100 ps resulting in 6071 conformations. This pruning has no significant effect on the computed averages, as shown in Supplementary Table S1, and thus does not substantially affect the conclusions. However, it makes the following analysis faster since it reduces the number of analyzed conformations. The reduced ensemble was then used to calculate the relative average signals 〈*f*(*d*_*i*_)〉_*e*_/*f*(*d*_*i*,exp_).

The relative average signals of the overall ensemble violations are listed in Table 1, column RECT. As expected from the Lagrangian multipliers, the violations for pairs 14G_H8_–13U_H1′_, 14G_H8_–14G_H1′_, 15U_H6_–15U_H1′_, for which λ_*i*_ became positive during the course of the simulation, have disappeared and the relative average signals are now >1. Only for the unconverged 17A_H8_–16G_H8_, the relative average signal is still well below 1, however better than in the unrestrained MD. Additionally, there are violations for 12U_H6_–11G_H1′_, and close to the ends 25U_H6_–24A_H1′_ and 26G_H8_–25U_H6_.

### Reweighting

By construction, the NOE observables in the experimentally restrained simulation would converge to the correct regime with longer simulation time. Since the simulation has not converged, however, there were still NOE observables which violated the experimental thresholds, and therefore all snapshots were reweighted using the ME method. The statistical efficiency, which is calculated from the weights and shows how much of the ensemble has to be discarded to obtain the correct averages, was calculated from the weights as *s* = 0.71, which means that 29% of the snapshots are redundant. (In comparison, *s* = 0.002 when reweighting the unrestrained simulation, which corresponds to a very uneven distribution of weights with few snapshots highly overweighted.) We notice that, in principle, an infinitely long restrained simulation should lead to an exact agreement with experiment without the need for this additional reweighting step, at least if the restraints can be satisfied by physically possible structures. Thus, in this case, we can consider this final reweighting procedure as a way to correct for the finite length of the simulation.

In Supplementary Figure S7 we report the value of the statistical efficiency as a function of simulated time. Here, it can be appreciated that the statistical efficiency grows quickly to ≈0.9 in the first 210 ns, then slowly decreases to ≈0.7, in agreement with the behavior of the ensemble violations reported in Supplementary Figure S6. This initial part of the simulation is required in order to generate an ensemble compatible with experiment. In principle, most of the relevant structures are already sampled at this stage. We qualitatively discuss the results that are obtained for the ensemble up to 210 ns below. The following part of the simulation enriches the generated ensemble with additional structures, without decreasing the agreement with experiment, and thus the statistical efficiency, significantly. The small observed decrease is likely due to the fact that the schedule used to update the Lagrangian multipliers makes them fluctuate on timescales that are too long to be sampled. As a consequence, in order to obtain a better agreement with experiment one should run the simulation even longer. In any case, the final results are made compatible with experiment by the reweighting procedure. Monitoring the statistical efficiency upon reweighting in the course of the simulation is cheap, and might be used to guide the decision of when the simulation should be stopped.

### Clustering

The 6071 snapshots in the reweighted ensemble were clustered to understand which different conformations are present and at which population. In the pre-clustering, snapshots were grouped by their χ conformation, which resulted in 11 different χ conformations for the whole ensemble (Table 2). The distinction on the χ angle is done to avoid that NOE observables calculated for a cluster are dominated by singular *syn* structures. In pre-cluster 1, which covers 48% of the weighted ensemble, only residue 16 is in *syn*, which corresponds to the conformation in ensembles NMR1, NMR2 and MD. 30% of the ensemble had no residue in *syn* (pre-cluster 2). 14% of the ensemble had only residue 14 in *syn* and 6% had residues 14 and 16 in *syn* (pre-clusters 3 and 4). Residue 15 was found in *syn* only in the low-populated (<1%) pre-clusters 6, 7 and 9–11. Outside of residues 14–17, no residue with a *syn* glycosidic bond angle was found in the entire ensemble.

**Table 2.** χ conformations found in the 6071 snapshot RECT ensemble. In column χ conf., 1 corresponds to *syn* conformation, 0 to *non-syn*

#	χ conf. res. 13–17	% of RECT	Number of cl.	≤5 largest cl.
1	00010	48.0	91	1 2 3 7 8...
2	00000	29.5	143	4 5 10 11 16...
3	01000	13.6	80	6 9 13 21 22...
4	01010	5.7	43	12 15 29 30 56...
5	00011	1.4	6	19 61 164 190 243...
6	00100	0.7	15	69 115 120 201 240...
7	00110	0.5	6	68 87 136 273 336...
8	00001	0.3	3	62 312 318
9	01100	0.2	5	110 118 193 274 389
10	01110	3e-2	2	343 383
11	00111	2e-2	1	386

The subsequent use of a maximum-clique clustering, which results in clusters in which all pairwise eRMSDs are less than 0.7, also avoids the problem of having outliers in a cluster which could change the cluster average of the back-calculated NOE signal considerably, due to the $d_{ij}^{-6}$ dependence. The threshold of 0.7 was chosen since it was shown that two conformations with an eRMSD below that value have an essentially identical map of base-base contacts as defined using the Leontis–Westhof classification (46,47).

The clustering resulted in 395 clusters, of which 105 contained only one structure. The correspondence between the pre-clusters and the largest clusters is shown in Table 2.

**Figure 1.**
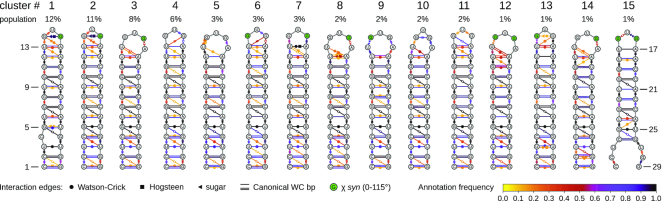
Dynamic secondary structure representation of the 15 largest clusters. Symbols represent interactions as defined by the Leontis–Westhof classification. The colors represent the frequency of the annotations per cluster. Residues with the glycosidic bond angle in *syn* are shown in green.

**Figure 2.**
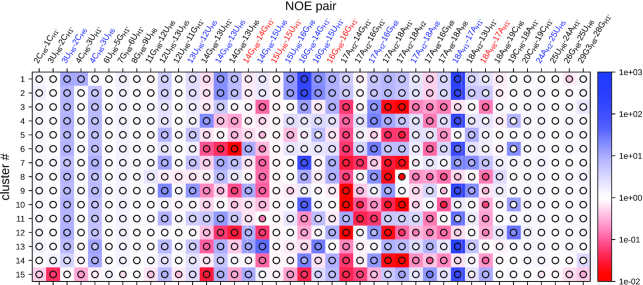
Relative ensemble averages (squares) and relative ensemble medians (circles) for the 15 largest clusters in the RECT ensemble. Red colors indicate values below 1, so the experimental NOE thresholds are violated. Blue colors indicate agreement with the experimental data.

Sixty-nine clusters had a population of >0.2% (sum of weights of the structures in the cluster), and a minimum number of 11 structures each. The secondary structure representation of the annotated clusters is shown in Figure 1 for clusters 1–15, which together represent 60% of all snapshots. The colors of the annotations indicate their population within each cluster. Cluster #2 is equivalent to the most prominent conformation in the unrestrained MD simulation. In the 15 largest clusters, residues 14 and 16 appear in both *syn* (green background) and *non-syn* conformation, while residue 15 is always in *non-syn*. Figure 2 shows which NOE thresholds are violated in which of the 15 largest clusters. The background color of each square indicates the relative cluster average 〈*f*(*d*_*i*_)〉_*wc*_/*f*(*d*_*i*,exp_), while the color inside the circle corresponds to the relative cluster median med[*f*(*d*_*i*_)]_*cl*_/*f*(*d*_*i*,exp_), i.e. the point with an equal amount of signals above and below. If the cluster average is not dominated by single, very high values, the two values should be in the same range. This is the case for most averages, only in a few cases the median is very close to the threshold (med[*f*(*d*_*i*_)]_*cl*_/*f*(*d*_*i*,exp_) = 1), but the average is clearly above. This confirms that structures belonging to the same cluster are homogeneous for what concerns their capability to explain each data point. For the proton pair 15U_H6_–15U_H1′_, which is related to χ_15_, the NOE observable requires structures from clusters smaller than the 15 most populated ones to be satisfied. The all-atom coordinates of the 69 most populated clusters are included in PDB format as Supplementary Material.

Supplementary Table S2 shows the indices of the clusters where the critical NOE thresholds in the loop are satisfied. For the intra-residue pair 14G_H8_–14G_H1′_, all clusters which satisfy the threshold have residue 14 in *syn* conformation. The same holds for 15U_H6_–15U_H1′_, which is satisfied only in the smallest clusters 68 and 69. The thresholds for 14G_H8_–14G_H1′_ and 14G_H8_–13U_H1′_ are never satisfied simultaneously in the same cluster, since they require mutually exclusive *syn* and *anti* conformations of residue 14, respectively.

### Minimal sets of clusters

For the 69 clusters with population $>0.2\%$, the minimal number of clusters which can be reweighted such that their overall averages satisfy all NOE restraints is 4. In particular, we are interested in constructing subsets of clusters that are as simple as possible to interpret (that is, containing the smallest possible number of structures) but at the same time that correspond to populations as similar as possible to those obtained by the reweighted RECT simulation. The sets have thus been sorted by the Kullback–Leibler divergence between the new weights and the original weights of the clusters. Table 3 shows the 10 sets with the lowest Kullback–Leibler divergences, i.e. those which have the highest agreement with the distribution sampled by the force field.

**Table 3.** Each line represents a set of clusters which, with the population in parenthesis, agree with all experimental data. They have been ordered by the Kullback–Leibler divergence *D*_*KL*_ between the given populations and the original populations of these clusters

c1	c2	c3	c4	*D*_*KL*_
2 (50%)	4 (27%)	9 (21%)	69 (2%)	1.72
1 (42%)	4 (30%)	9 (25%)	69 (3%)	1.72
2 (50%)	4 (27%)	9 (22%)	68 (1%)	1.72
1 (44%)	4 (28%)	9 (27%)	68 (1%)	1.72
2 (71%)	9 (17%)	11 (11%)	69 (1%)	1.92
2 (70%)	9 (18%)	11 (11%)	68 (1%)	1.92
2 (74%)	9 (19%)	26 (5%)	69 (2%)	2.00
2 (73%)	9 (20%)	26 (5%)	68 (2%)	2.00
2 (75%)	9 (20%)	37 (3%)	69 (2%)	2.02
2 (74%)	9 (21%)	37 (3%)	68 (2%)	2.02

**Figure 3.**
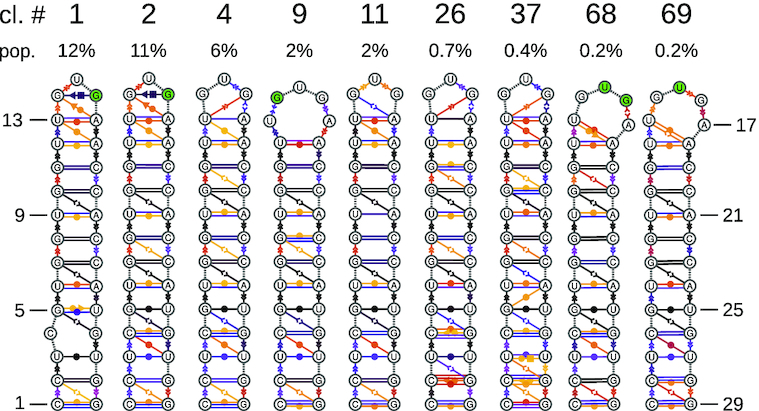
Clusters from Table 3. The right combination of at least four of them with the right population suffices to satisfy all experimental data. Annotation symbols and colors are identical to Figure 1.

**Figure 4.**
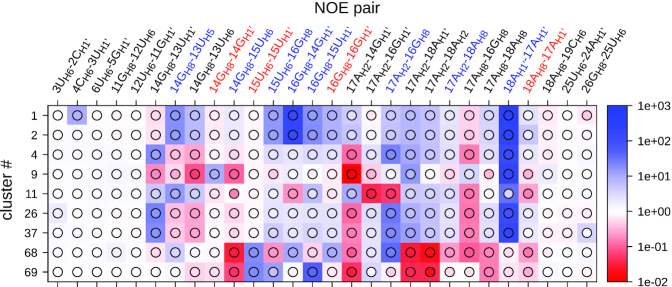
Relative ensemble averages (squares) and relative ensemble medians (circles) corresponding to the clusters in Table 3 and Figure 3. Colors as in Figure 2.

**Figure 5.**
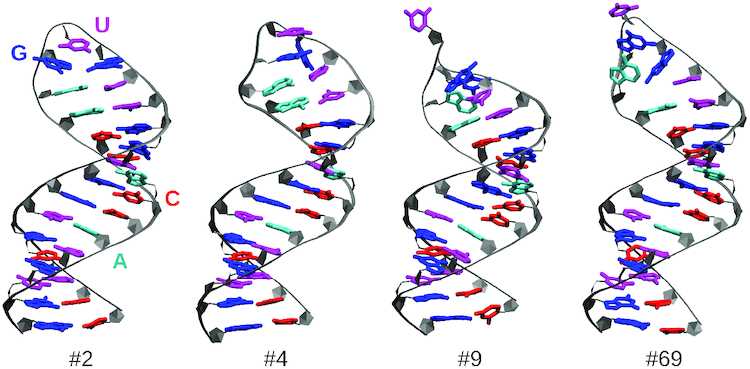
Three-dimensional representation of four representative clusters that are sufficient to satisfy all experimental data. The cluster numbers are consistent with those reported in Table 3.

Figure 3 shows the secondary structures of the 9 clusters from Table 3, and Figure 4 the relative cluster averages and the relative cluster means for pairs in which the average is below the threshold at least once. In each set, one of clusters 1 and 2 is present, which have a very similar structure in the loop region and satisfy most of the NOE thresholds. Each set has cluster 9, which has residue 14 in *syn* conformation and satisfies the strong NOE restraint 14G_H8_–14G_H1′_, and the medium restraint for pair 17A_H8_–16G_H8_. Interestingly, cluster 9 has 17A unpaired. This residue was observed to be methylated by dimethyl sulfate in biologically active construct containing this hairpin (16), providing an indirect confirmation of the presence of these structures in the ensemble. Each set also has one of clusters 68 and 69 at a small population, which both have residue 15 in *syn* conformation and therefore satisfy the strong NOE restraint for pair 15U_H6_–15U_H1′_. The remaining clusters 4, 11, 26 and 37 have all residues in *anti/high-anti* conformation and satisfy the NOE restraint for pair 14G_H8_–13U_H1′_. A 3D representation of a set of representative clusters is reported in Figure 5.

Supplementary Figure S8 shows the results of the same analysis for the ensemble with the highest Kish’s sample size, up to 210 ns. As a result of the clustering algorithm, the clusters are not identical, however some clusters very similar to the essential clusters from Table 3 end up in the list of best sets (Supplementary Figure S8A), as can be seen by comparison of Figure 3 with Supplementary Figure S8B, respectively Figure 4 with Supplementary Figure S8C. The best set in the full ensemble (clusters 2, 4, 9, 69) corresponds to the set of clusters 1, 12, 4 and 34 in the shorter ensemble. Notably, for the short ensemble one reweightable set of only three clusters could be also found, which satisfies the experimental restraints. However, this set involves two clusters with very low ensemble populations (cluster 22: 0.5% and cluster 39: 0.2%) and has a high Kullback–Leibler divergence with respect to the sets with four clusters. We conclude that, for the shorter ensemble, the results are qualitatively the same and a set of four conformations containing conformations with residue 14, 15 and 16 in *syn* is needed to satisfy all restraints. The larger the ensemble, however, the more structures are contained in one cluster and the more robust are the computed cluster averages, avoiding overestimation of low-populated clusters that happen to satisfy a certain set of restraints.

### PDB search

**Figure 6.**
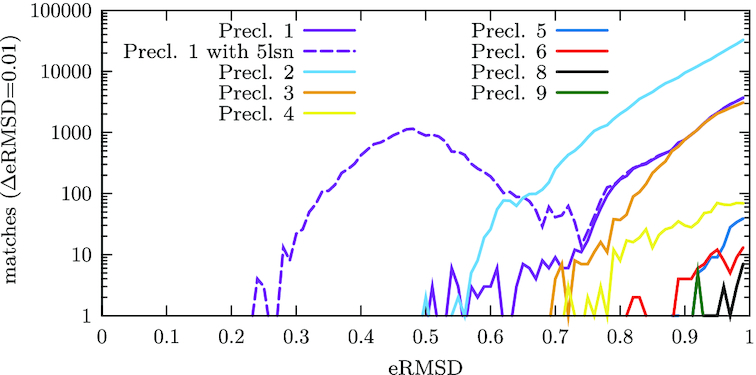
Number of matches by pre-cluster. The violet dashed line respresents the matches for pre-cluster 1 including those matching the deposited NMR structure #5lsn. For pre-clusters 7, 10 and 11, no matching motifs were found.

All RNA-containing structures in the PDB database have been searched for sequence-independent motifs similar to the 6071 conformations contained in the RECT ensemble. In 1173 out of the 4572 RNA-containing PDB database structures (some of which contain multiple models), 6052 fragments were found with eRMSD <1.0 and matching χ conformation, resulting in a total of 305 442 pairs of matching PDB fragments and sampled snapshots. Figure 6 shows the number of matches per pre-cluster (same χ conformation, compare Table 2). 79% of the motif matches match pre-cluster 2, which has no residue in *syn* conformation, in agreement with the low population of *syn* conformation observed in the PDB. 13% match pre-cluster 1, where residue 16 is in *syn*. These matches include the matches with the deposited PDB structure #5lsn, included in the dashed line in Figure 6. 7% match pre-cluster 3 with residue 14 in *syn*. Out of the pre-clusters with the central residue 15 in *syn*, only pre-clusters 6 (residue 15 in *syn*) and 9 (residues 14 and 15 in *syn*) have a few matches (89 resp. 5 matches).

The best matching motifs from the PDB database for each pre-cluster are listed in Table 4 (matches for #5lsn are excluded). The upper part shows the motifs with the best match (minimum eRMSD) to any of the structures in the pre-cluster, while the lower part shows the motifs which have the most matches per pre-cluster, together with the minimum eRMSD. For pre-clusters 10 and 11, which both have residues 15 and 16 in *syn*, no matching structures have been found in the PDB database. Only for pre-clusters 1–3, matches with eRMSD<0.7 have been found, which means that the structural annotations in query and match are essentially identical. Supplementary Table S3 gives more details about these matches.

**Table 4.** Best matches by pre-cluster (matches for the PDB ID #5lsn are excluded). The fourth column (*n*_matches_) shows the number of structures in the pre-cluster which match the motif. The number in parenthesis is the matching percentage of the pre-cluster

Best match in pre-cluster					
#	PDB	Position	*n*_matches_	eRMSD	Molecule
1	5zet	A:C2087	675 (23%)	0.51	23S rRNA
2	3j9w	bdl 1, A:C1457	295 (16%)	0.51	16S rRNA
3	4tue	bdl 2, A:C610	131 (16%)	0.70	23S rRNA
4	4v70	bdl 2, A:C2794	119 (34%)	0.72	23S rRNA
5	4u56	bdl 3, d:G776	25 (30%)	0.89	18S rRNA
6	5wsg	E:A9	1 (2%)	0.82	SNR6 snRNA
8	5xxu	2:A1353	3 (18%)	0.89	18S rRNA
9	5o61	bdl 2, A:C1008	1 (8%)	0.92	16S rRNA
Motif with most matches in pre-cluster					
#	PDB	Position	*n*_matches_	eRMSD best	Molecule
1	2n4l	A24, M7	1612 (55%)	0.65	HIV-1 intron splicing silencer
2	1hr2	B:A233	673 (38%)	0.69	group 1 intron
3	4v5d	bdl 1, A:G836	231 (28%)	0.75	16S rRNA
4	4v70	bdl 2, B:G84	123 (35%)	0.76	5S rRNA
5	4u56	bdl 3, d:G776	25 (30%)	0.89	18S rRNA
6	5juo	bdl 1, A:C449	5 (11%)	0.90	18S rRNA
8	5xxu	2:A1353	3 (18%)	0.89	18S rRNA
9	5o5j	A:C1008	1 (8%)	0.92	16S rRNA

Position nomenclature: bdl #1 C:R#2, bundle number #1, chain C, residue R, residue number #2, model M.

Supplementary Table S4 shows the matches for the best-set clusters from Table 3. Out of those, matches with essentially identical conformation, i.e. eRMSD <0.7, could be found only for clusters 2 (PDB #2n4l, min. eRMSD 0.65) and 11 (PDB #3ccr, min. eRMSD 0.59, and PDB #1q7y, min. eRMSD 0.62). Cluster 2 belongs to pre-cluster 1 with residue 16 in *syn*, cluster 11 to pre-cluster 2 with no residue in *syn*. For the other relevant clusters 9, 68 and 69, which have residues 14 or 15 in *syn*, only matches with higher eRMSD could be found. Notice that PDB #2n4l has been already identified as a match in (16) since its structure is similar to the deposited structure (#5lsn). Importantly, the matches corresponding to the low population clusters were not reported previously and could only be identified thanks to the enhanced sampling simulation generating those clusters.

Since it is known that SINEB2 acts as a translation enhancer, the PDB matches in ribosomes were separately analyzed. Out of the 6052 matching positions, 5117 (85%) have been found in ribosomal structures. The best matches with eRMSD <0.7 are given in the Supplementary Material. These ribosomal structures have been annotated to find basepairs between the matching loop and other regions of the ribosome. Forty seven matches have at least 3 bp with another single stranded region of the ribosome (data not shown). When replacing the sequence of the match with the SINEB2 sequence, however, these basepairs are not conserved.

## DISCUSSION

RNA molecules are highly flexible and can exhibit multiple metastable states that are functionally relevant. In these cases, a diverse set of conformations is needed to explain and to reproduce solution experiments. In this work, we showed how enhanced sampling techniques in MD simulations can be synergistically combined with NMR experimental restraints to obtain such a diverse ensemble and how results can be analyzed so as to be interpreted in terms of a reduced number of molecular conformations.

Sampling was enhanced using a replica-exchange scheme, which has the disadvantage that kinetic information is lost, due to the frequent exchange of conformations between simulations under different conditions. However, it has two strong advantages: First, the conformational space sampled in unbiased MD simulations is too narrow, i.e. the conformations too similar to each other, such that an *a posteriori* ME reweighting as we used it here cannot produce new conformations, but will only heavily overweight the few conformations which agree most with the experimental restraints (16,22,48). Second, when using an ME correction on-the-fly during an unbiased simulation, this correction to the force field might not be effective in facilitating the necessary conformational transitions, since e.g. in our case, the forward model for the NOEs is related to the inter-proton distance and not to the glycosidic bond angle χ, even though some NOEs are clearly related to this angle. Using replica exchange with a smart choice of collective variables allows the simulation to overcome energetic barriers and to sample a heterogeneous set of conformations, while the combination with ME restraints helps to adjust the populations of these conformations to an ensemble compatible with the experimental data. Nevertheless, a final ME reweighting is needed to obtain a perfectly compatible ensemble to compensate for the limited simulation time. The statistical efficiency of such reweighting might even be used to decide if the simulation length is sufficient.

The combination of enhanced sampling, adaptive ME restraints during the simulation, and *a posteriori* ME reweighting allows to generate an ensemble compatible with the experiment, using as little extra information to the accurate force field and state-of-the-art enhanced sampling simulation as possible. The simulation cannot replace the experiment, but generates a plethora of extra conformational details and information. While, in this specific case, the existence of metastable *syn* states could have been guessed directly from the NOE data, the mutual incompatibility of experimental data in some of the alternative structures is a genuine result from the MD protocol.

The ensemble is then simplified into clusters to be interpreted directly. In the MP step, these clusters are grouped into small sets, and the presence of each of the clusters is justified by at least one of the experimental signals. This is particularly valuable since it allows to double check experimental datapoints which are necessary to justify unexpected structures.

The obtained clusters are then compared to the PDB to verify if similar structures are already known. Whereas most of the matches are cryo-EM structures which are probably not accurate enough to be used as a reference, some accurate X-ray structures were also found.

Interestingly, most of the matches are in ribosomal RNA. This might be related to the SINEB2 function as translation enhancer, as this might be mediated by interactions with the ribosome. We could not find any ribosomal position, where the matched loop basepairs with the environment when replacing the sequence with the SINEB2 sequence. Such a match could have been a hint where and how SINEB2 interacts directly with the ribosome. However, the interaction could also be steric, not involving basepairs. It would be interesting to see if future experiments reveal any interactions involving the matching positions.

On one hand, the structural context has been shown to be capable to stabilize high-energy structures, so that fragments extracted from the PDB agree well with solution experiments for both proteins (49) and RNA systems (50). On the other hand, the low-population structures identified in this work (structures with residue 15 in *syn*) were not present in the PDB and could only be found thanks to a synergistic combination of MD simulations and NMR experiments. *Syn* bases have been shown to be prevalent in active sites of functional RNAs (51).

The approach shown here could be effectively used to model at atomistic detail low-population states that are often suggested by NMR data (10). In this work, we applied state-of-the-art enhanced sampling combined with ME experimental restraints, both as on-the-fly restraints and as *a posteriori* reweighting, in order to reconstruct the conformational ensemble of a 29 nt RNA hairpin. We found that some conformations needed to satisfy the experimental data imply mutually incompatible NOE restraints, and that at least four different conformations are needed to completely satisfy the experimental data. Our protocol could be used for future analysis of the conformational ensemble of molecules where NMR data indicate the existence of low-populated states.

## Supplementary Material

gkz1184_Supplemental_Files
