# Supplementary material for: Conformational ensembles of an RNA hairpin using molecular dynamics and sparse NMR data
Source: Nucleic Acids Res. 2019 Dec 31;48(3):1164–74. doi: 10.1093/nar/gkz1184 (PMC7026608; doi:10.1093/nar/gkz1184)
Supplement: gkz1184_Supplemental_Files [file gkz1184_supplemental_files.zip › sm.pdf]

Conformational ensembles of an RNA hairpin using molecular dynamics and sparse NMR data

Supplementary Material

Sabine Reißer[1,*], **Silvia Zucchelli**[1,2], **Stefano Gustincich**[3], **Giovanni Bussi**[1,*]

[1]Scuola Internazionale Superiore di Studi Avanzati (SISSA), Via Bonomea 265, 34136 Trieste, Italy
[2]Department of Health Sciences, Center for Autoimmune and Allergic Diseases (CAAD) and Interdisciplinary Research Center of Autoimmune Diseases (IRCAD), University of Piemonte Orientale, Novara, Italy
[3]Central RNA Laboratory and Department of Neuroscience and Brain Technologies, Istituto Italiano di Tecnologia (IIT), 16163, Genova, Italy

List of Tables

S1 Effect of RECT ensemble reduction . . . 4
S2 Critical NOEs for the RECT ensemble . . . 4
S3 Details for PDB matches for preclusters 1-3 . . . 4
S4 PDB matches for clusters from best minimal sets . . . 5

List of Figures

S1 Secondary structures of PDB #5lsn and of RECT starting structures . . . 6
S2 Glycosidic bond angles in the loop, from unrestrained MD simulation . . . 7
S3 χ vs. distance distribution from RECT simulation . . . 7
S4 Effective Lagrangian multipliers in RECT simulation . . . 7
S5 RECT simulation convergence . . . 8
S6 NOE ensemble violations over RECT simulation runtime . . . 8
S7 Kish's effective sample size over simulation runtime . . . 8
S8 Best sets for 210 ns ensemble . . . 9

*To whom correspondence should be addressed. Email: bussi@sissa.it, Sabine.Reisser@mdc-berlin.de

© The Author(s)
This is an Open Access article distributed under the terms of the Creative Commons Attribution Non-Commercial License (http://creativecommons.org/licenses/by-nc/2.0/uk/) which permits unrestricted non-commercial use, distribution, and reproduction in any medium, provided the original work is properly cited.

RECT SIMULATIONS

RECT simulations were performed using 8 replicas in a replica exchange scheme. Within each replica, 6 collective variables were biased using well-tempered metadynamics. In particular, for each of the three apical nucleobases (G14, U15, and G16) we biased:

- The χ glycosidic bond angle.

- The coordination number between the center of mass of the nucleobase and the centers of mass of all the other nucleobases in the hairpin. The coordination number was computed as a sum of switching functions in the form $\sum_i \frac{1}{1+\left(\frac{d_i}{r_0}\right)^6}$ where $r_0 = 4\mathring{A}$ and d_i is the distance with the center of mass of the i-th nucleobase.

Well-tempered metadynamics was used to concurrently bias those six variables with a bias factor chosen with a geometric progression between 1 (first replica) and 8 (last replica). The first replica thus reports unbiased statistics, but its sampling is enhanced by the exchanges with the other replicas. Only the first replica was then analyzed. This protocol is equivalent to the one used in the paper where RECT was proposed (1).

NOE RESTRAINTS

According to the maximum entropy principle, the correcting potential should have the form

$$\sum_i \frac{\lambda_i}{d_i^6}$$

where d_i is the proton-proton distance corresponding to a given peak in the NOE spectrum. The Lagrangian multipliers λ_i should be adjusted in order to enforce the ensemble average. Following Ref. (2), we here adjusted the λ_i multipliers based on the discrepancy between the experimental signal, computed as $\frac{1}{3.6^6}\text{Å}^{-6}$, $\frac{1}{5.0^6}\text{Å}^{-6}$, and $\frac{1}{6.5^6}\text{Å}^{-6}$ for strong, medium and weak signals respectively, and the instantaneous one, computed as $\frac{1}{d_i^6}$. The instantaneous value was only collected in the reference replica and not in the biased ones. The NOE restraints acting on the biased replicas were computed by dividing the Lagrangian multipliers optimized on the reference replica by the corresponding bias factor. This choice results in a high acceptance in the replica exchange procedure but at the same time allows biased replicas to violate easily the restraints, thus accelerating sampling. The restraints were only applied when for negative values of λ.

CALCULATION OF TIME-DEPENDENT VIOLATIONS

The relative average signal $f_{rel,i}$ for an ensemble of structures e is defined as

$$f_{rel,i} = \langle f(d_i) \rangle_e / f(d_{i,exp}) \qquad \text{(S1)}$$

The number of NOE observables $N_{NOE} = 125$.

Now, the time-dependent ensemble violation is calculated for the ensemble from the simulation time interval [0,t]:

$$f_{rel,i}(t) = \langle f(d_i) \rangle_{[0,t]} / f(d_{i,exp}) \tag{S2}$$

$$\text{viol}_{ens}(t) = \sum_{i=1}^{N_{NOE}} max(0, 1 - f_{rel,i}(t)) \tag{S3}$$

The instantaneous violations are not calculated for an ensemble, but for each snapshot (conformation) seperately:

$$f_{rel,i,inst}(t) = f(d_i,t) / f(d_{i,exp}) \tag{S4}$$

$$\text{viol}_{inst}(t) = \sum_{i=1}^{N_{NOE}} max(0, 1 - f_{rel,i,inst}(t)) \tag{S5}$$

The time-dependent ensemble violations and instantaneous violations are shown in Fig. S6.

EFFECT OF RECT ENSEMBLE REDUCTION

In principle, due to the d^{-6} dependence of the Nuclear Overhauser Effect (NOE) signals, single conformations with extra-short distances can have significant impact on the average NOE, i.e. the reduction of the time-resolution of snapshots might decrease the average NOEs calculated from the snapshots, since short-distance conformations might now be missing.
However, in reality we observe that the NOE averages in the dt=1ps ensemble are essentially identical to the dt=100ps ensemble, the maximum difference being 5%. Proton pairs where the averages differ by more than 1% and which are in the critical region around the threshold are shown in Table S1.

RRNA MOTIF MATCHES FROM THE PDB

The best matches from the sequence-independent PDB motif search, which have eRMSD<0.7 and come from ribosomal structures, are found in file `PDB_04-10-2019_rRNA_matches`. Columns are:

```
pdb            PDB ID (including bundle)
resname        [A,U,C,G]
chain          chain (in given bundle)
resid          residue ID in PDB file where the 9nt match starts
min_ermsd      minimum eRMSD between the match and any structure from the RECT ensemble
n_matches      number of matches in the RECT ensemble
chi_conf       chi conformation for the loop (nt #11-19); 0: not syn, 1: syn
```

Table S1. RECT simulation: proton pairs with NOE observables with more than 1% deviation between dt=1ps and dt=100ps trajectory and 0.7< $f_{rel,i}$ >1.3. The relative average signal for NOE i is $f_{rel,i} = \langle f(d_i)\rangle_e / f(d_{i,exp})$. d_{exp} are the experimental maximum distances, $s = 3.6$ Å, $m = 5.0$ Å, $l = 6.5$ Å.For discussion of this table see section .

pair	d_{exp}	$1 - \frac{f_{rel,dt=1ps}}{f_{rel,dt=100ps}}$	$f_{rel,dt=1ps}$	$f_{rel,dt=100ps}$
$24A_{H2}$–$25U_{H5}$	l	0.02	1.23	1.26
$15U_{H6}$–$15U_{H1'}$	s	-0.04	1.16	1.12
$17A_{H2}$–$16G_{H1'}$	m	0.04	1.19	1.24

Table S2. Critical NOEs with $\langle f(d_i)\rangle_e / f(d_{i,exp}) < 1.2$ for RECT ensemble (from Table 1 in the main text), in the loop region from residue 11 to 19, together with the clusters where they are satisfied clearly (relative average signal > 1.2, or weakly (relative average signal < 1.2 but > 1). Shown are the ids of the 3 largest clusters and in brackets the total number of clusters.

pair	d_{exp}	$\frac{\langle f(d_i)\rangle}{f_{i,exp}}$ RECT	clearly satified in cl # (n_{tot})	weakly satisfied in cl # (n_{tot})
$11G_{H8}$–$12U_{H6}$	m	1.14	3 7 8 (22)	4 6 10 (23)
$12U_{H6}$–$11G_{H1'}$	m	**0.94**	9 20 23 (5)	1 2 5 (32)
$14G_{H8}$–$13U_{H1'}$	m	2.39	4 11 24 (12)	50 62 64
$14G_{H8}$–$14G_{H1'}$	s	2.45	6 9 12 (18)	
$15U_{H6}$–$15U_{H1'}$	s	1.16	68 69	
$17A_{H2}$–$16G_{H1'}$	m	1.19	2 4 5 (22)	20 33 40 (6)
$17A_{H8}$–$16G_{H8}$	m	**0.54**	5 7 9 (18)	42 50 60
$18A_{H8}$–$19C_{H6}$	m	1.16	3 7 8 (23)	10 11 14 (15)
$19C_{H6}$–$18A_{H1'}$	m	4.17	4 6 7 (22)	1 2 3 (33)

Table S3. Details of matches for preclusters 1-3, related to Table 4 in the main text.

PDB ID	pcl[a]	molecule	organism	method, resolution	ref
1hr2	2	mutant of a P4-P6 intron domain	*Tetrahymena thermophila*	X-ray	(3)
2n4l	1	HIV-1 Intron Splicing Silencer	HI Virus	NMR	(4)
3j9w	2	16S	*Bacillus subtilis*	cryo-EM 3.5-3.9Å	(5)
4v5d	3	16S	*Thermus thermophilus*	X-ray 3.5Å	(6)
5zet	1	23S	*Mycobacterium smegmatis*	EM 3.2Å	(7)
4tue	3	23S	*Thermus thermophilus*	X-ray 3.5Å	(8)

[a]Precluster match.

Table S4. Match overview for clusters from Table 3 from the main text (matches for the deposited NMR structure 5lsn are excluded). Position nomenclature: bdl #1 C:R#2, bundle number #1, chain C, residue R, residue number #2, model M.

Best match in cluster						
#	pcl	PDB	position	$n_{matches}$[a]	eRMSD	molecule
1	1	2n4l	A:A24, M7	747 (100%)	0.72	HIV-1 intron splicing silencer
2	1	2n4l	A:A24, M7	641 (100%)	0.65	HIV-1 intron splicing silencer
4	2	1i3x	A:G6, M11	387 (98%)	0.75	23S rRNA loop
9	3	4pqv	A:A56	50 (66%)	0.77	flavivirus
11	2	3ccr	0:G116	109 (99%)	0.59	23S rRNA
26	2	1i3x	A:G6, M11	45 (96%)	0.79	23S rRNA loop
37	2	1i3x	A:G6, M3	25 (93%)	0.81	23S rRNA loop
68	7		—			
69	6	5juo	bdl 1, A:C449	3 (27%)	0.90	18S rRNA

Motif with most matches in cluster						
#	pcl	PDB	position	$n_{matches}$[a]	eRMSD best	molecule
1	1	2n4l	A:A24, M7	747 (100%)	0.72	HIV-1 intron splicing silencer
2	1	2n4l	A:A24, M7	641 (100%)	0.65	HIV-1 intron splicing silencer
4	2	1i3x	G6, M11	387 (98%)	0.75	23S rRNA
9	3	6gzq	bdl 1, c:C636	67 (88%)	0.77	23S rRNA
11	2	1q7y	A:G116	110 (100%)	0.62	23S rRNA
26	2	1i3x	A:G6, M3	46 (98%)	0.83	23S rRNA loop
37	2	1i3x	A:G6, M11	26 (96%)	0.81	23S rRNA loop
68	7		—			
69	6	5juo	bdl 1, A:C449	3 (27%)	0.90	18S rRNA

[a] Number of matched structures per cluster. In parenthesis percentage of cluster.

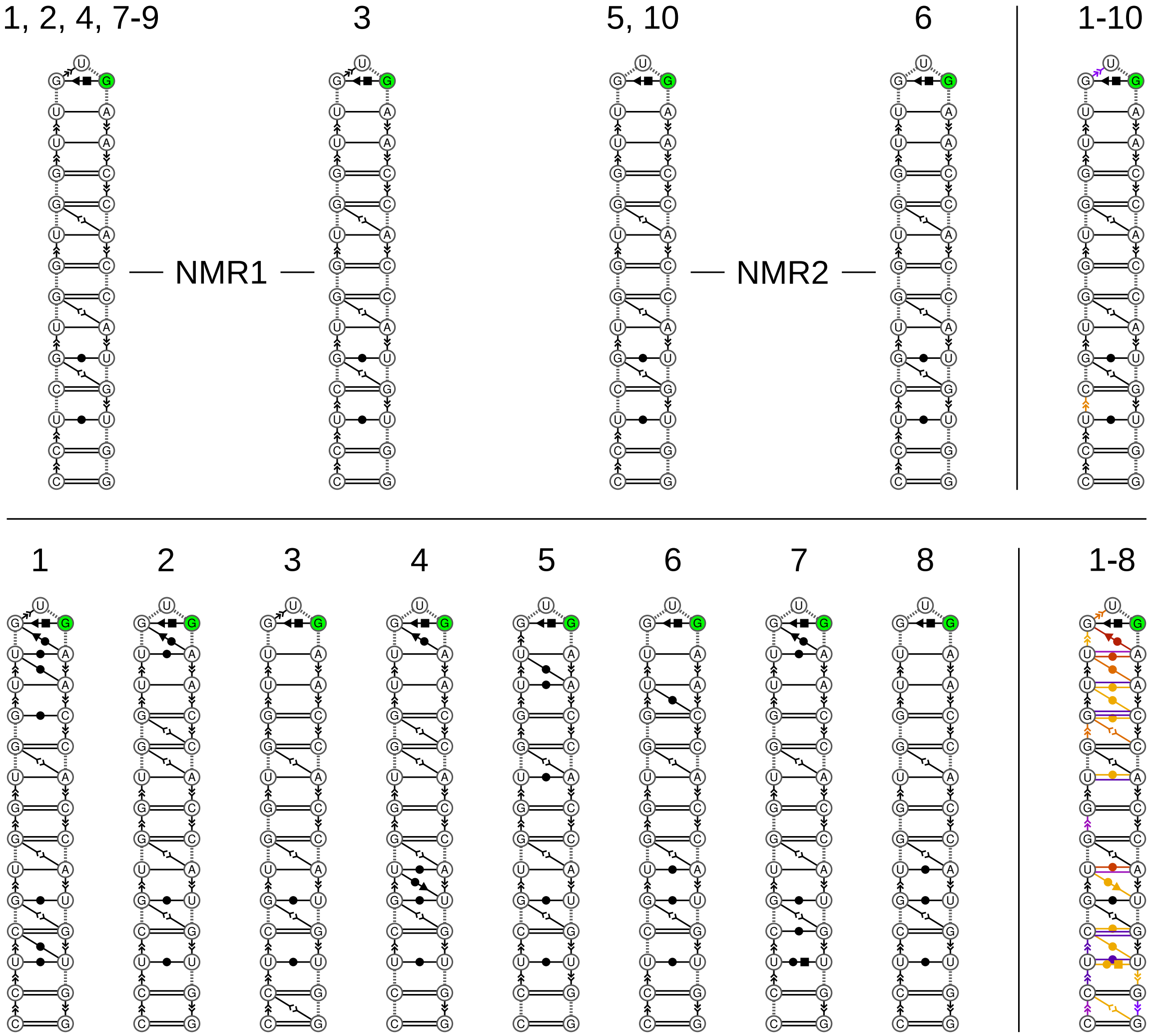

Figure S1. Top: Secondary structures of the deposited PDB structure #5lsn. On the right, all structures are combined in one figure, the color indicating the frequency of the annotation, from yellow (rare) to black (always; for symbol and color legends see Fig. 1 in the main text). Bottom: Secondary structures of the starting conformations for the 8 replicas. On the right all starting structures are combined in one figure.

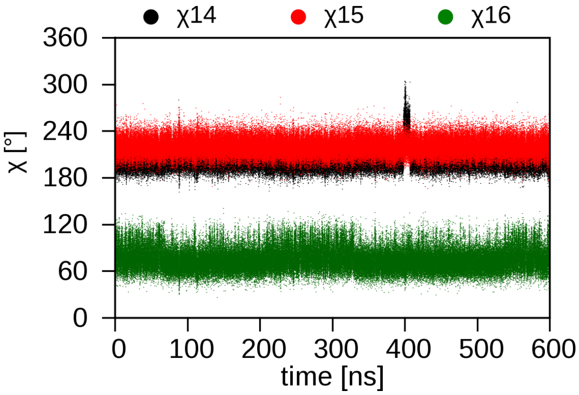

Figure S2. Evolution of χ_{14}, χ_{15} and χ_{16} during the unrestrained MD simulation.

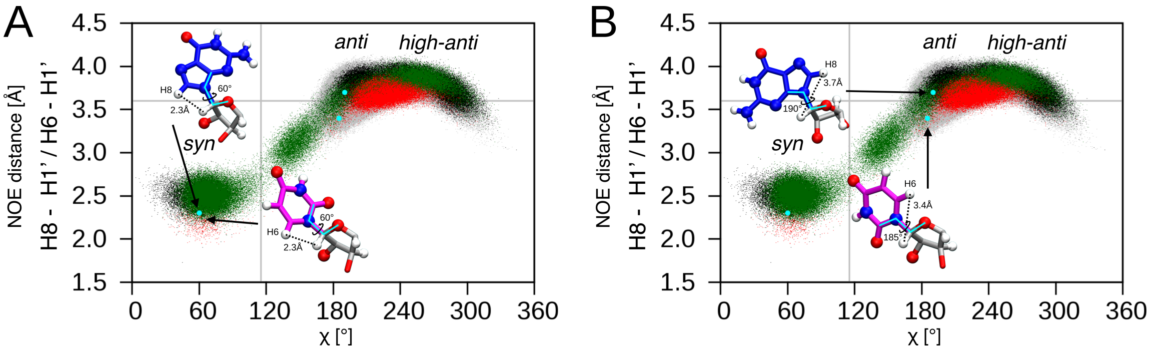

Figure S3. H8/H6-H1' distance over glycosidic bond angle, each dot represents one snapshot from the RECT simulation, for residue 14 (black), residue 15 (red), residue 16 (green), and all other residues (grey). Vertical line indicates 115°, the threshold for distinguishing *syn/anti* conformations. The horizontal line indicates the NMR threshold used for a strong signal, 3.6Å. A: Typical conformation of guanine (blue) and uridine (magenta) in *syn*, with respective distance and χ angle. B: Typical conformation of G and U in *anti*, with respective distance and χ angle.

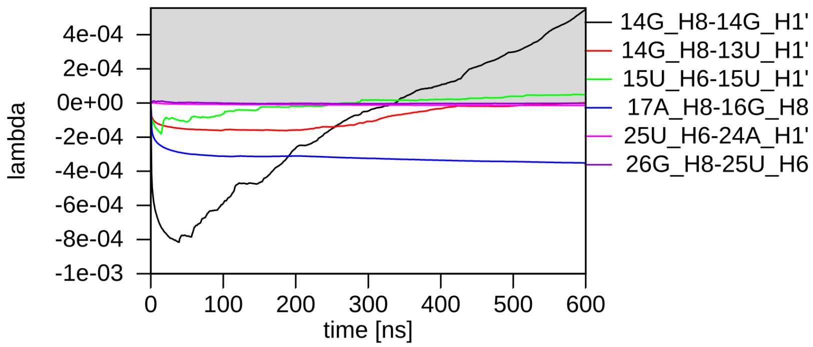

Figure S4. Lagrangian multipliers in the RECT simulation. Shown are only the ones which have values <-3e-6 when t>50ps

.

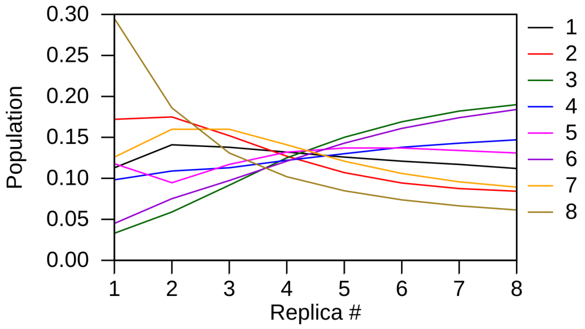

Figure S5. RECT simulation. Normalized amount of time spent in each replica for continuous trajectories with starting structures #1-8

.

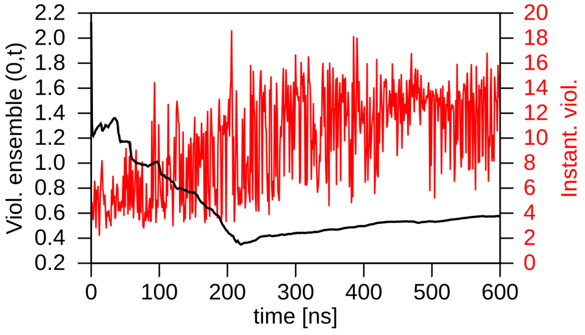

Figure S6. RECT simulation, ensemble violations viol$_{ens}(t)$ (ensemble from 0-t with dt=1ps; black curve) and instantaneous violations (viol$_{inst}(t)$, red curve). The red curve exhibits discontinuities when replica exchanges are accepted. Calculation of violations is explained above.

.

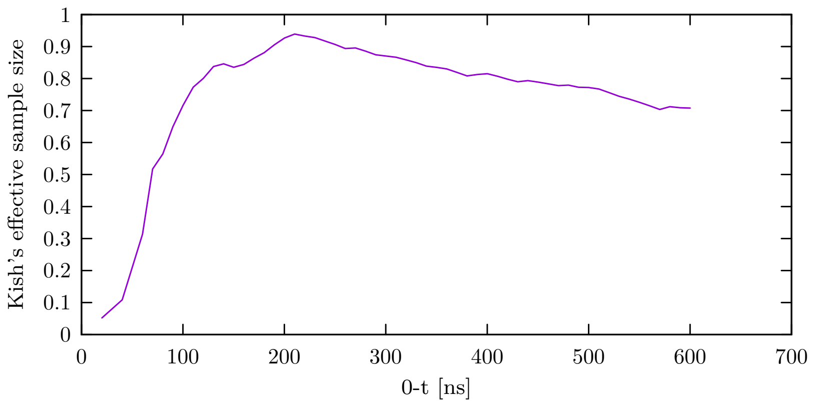

Figure S7. Kish's effective sample size as a function of RECT simulation time

.

A

c1	c2	c3	c4	D_{KL}
1 (36%)	22 (53%)	39 (11%)	-	3.03
1 (70%)	3 (22%)	12 (4%)	39 (4%)	1.03
1 (72%)	3 (21%)	28 (3%)	39 (4%)	1.07
1 (70%)	4 (21%)	12 (5%)	26 (4%)	1.12
1 (72%)	4 (20%)	26 (5%)	28 (3%)	1.16
1 (65%)	4 (25%)	12 (5%)	34 (5%)	1.19
1 (67%)	4 (24%)	28 (3%)	34 (6%)	1.24
1 (75%)	8 (14%)	12 (5%)	39 (6%)	1.24
1 (77%)	8 (14%)	28 (3%)	39 (6%)	1.28
1 (71%)	5 (11%)	12 (5%)	26 (13%)	1.30
1 (56%)	4 (38%)	12 (3%)	39 (3%)	1.32

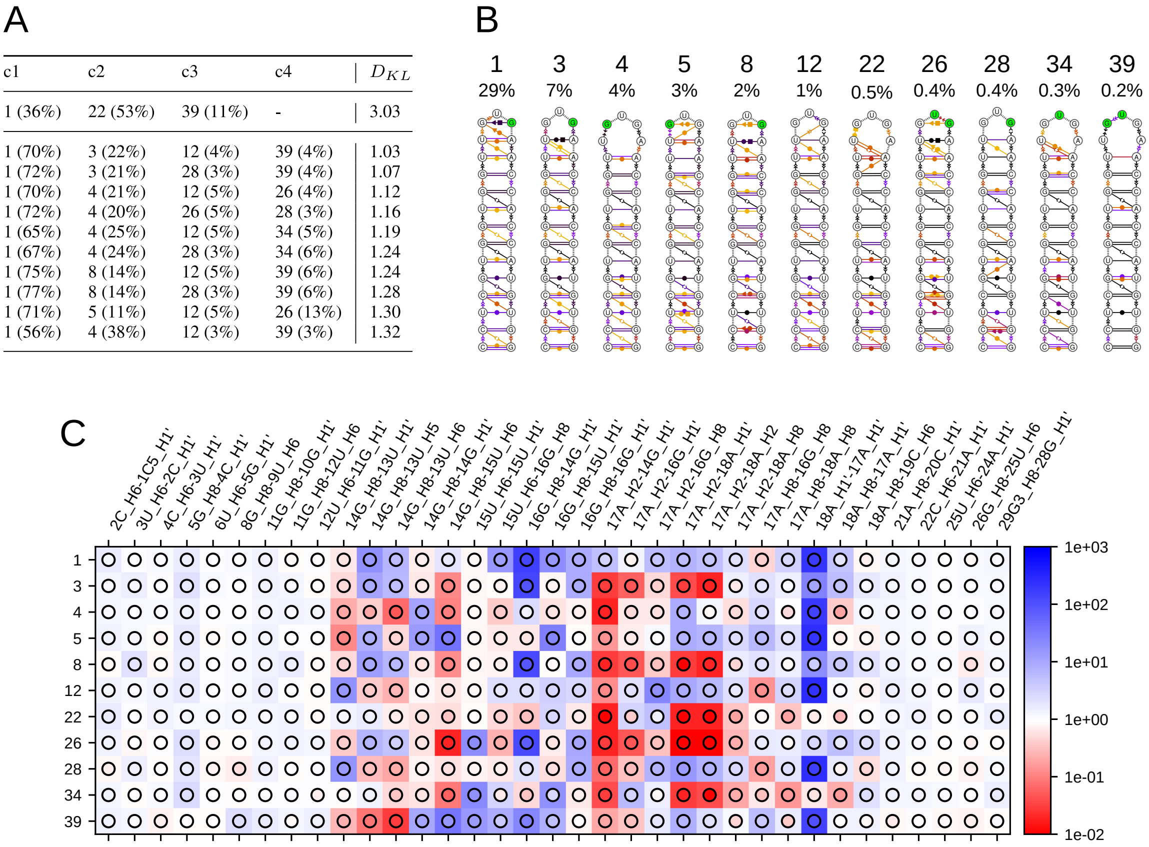

Figure S8. A: 1st line: set of 3 clusters which can be reweighted to satisfy all restraints. Below: best 10 sets (by lowest Kullback-Leibler divergence) of 4 clusters. B: dynamic secondary structures of clusters in A (Annotation symbols and colors are identical to Figure 1). Relative ensemble averages (squares) and relative ensemble medians (circles) corresponding to the clusters in A (colors as in Figure 2).

.

REFERENCES

1. Gil-Ley,A. and Bussi,G. (2015) Enhanced Conformational Sampling Using Replica Exchange with Collective-Variable Tempering. *J. Chem. Theory Comput.,* **11**(3), 1077–1085.
2. Cesari,A., Gil-Ley,A., and Bussi,G. (2016) Combining Simulations and Solution Experiments as a Paradigm for RNA Force Field Refinement. *J. Chem. Theory Comput.,* **12**(12), 6192–6200.
3. Juneau,K., Podell,E., Harrington,D.J., and Cech,T.R. (2001) Structural basis of the enhanced stability of a mutant ribozyme domain and a detailed view of RNA–solvent interactions. *Structure (London, England : 1993),* **9**(3), 221–31.
4. Jain,N., Morgan,C.E., Rife,B.D., Salemi,M., and Tolbert,B.S. (2016) Solution Structure of the HIV-1 Intron Splicing Silencer and Its Interactions with the UP1 Domain of Heterogeneous Nuclear Ribonucleoprotein (hnRNP) A1. *The Journal of biological chemistry,* **291**(5), 2331–44.
5. Sohmen,D., Chiba,S., Shimokawa-Chiba,N., Innis,C.A., Berninghausen,O., Beckmann,R., Ito,K., and Wilson,D.N. (2015) Structure of the Bacillus subtilis 70S ribosome reveals the basis for species-specific stalling. *Nature Communications,* **6**(1), 6941.
6. Voorhees,R.M., Weixlbaumer,A., Loakes,D., Kelley,A.C., and Ramakrishnan,V. (2009) Insights into substrate stabilization from snapshots of the peptidyl transferase center of the intact 70S ribosome. *Nature Structural & Molecular Biology,* **16**(5), 528–533.
7. Mishra,S., Ahmed,T., Tyagi,A., Shi,J., and Bhushan,S. (2018) Structures of Mycobacterium smegmatis 70S ribosomes in complex with HPF, tmRNA, and P-tRNA. *Scientific reports,* **8**(1), 13587.
8. Fagan,C.E., Maehigashi,T., Dunkle,J.A., Miles,S.J., and Dunham,C.M. (2014) Structural insights into translational recoding by frameshift suppressor tRNASufJ. *RNA (New York, N.Y.),* **20**(12), 1944–54.
